# The lncRNA Snhg11, a new candidate contributing to neurogenesis, plasticity and memory deficits in Down syndrome

**DOI:** 10.21203/rs.3.rs-3184329/v1

**Published:** 2023-09-25

**Authors:** Cesar Sierra, Miguel Sabariego-Navarro, Álvaro Fernández-Blanco, Sonia Cruciani, Alfonsa Zamora-Moratalla, Eva Maria Novoa, Mara Dierssen

**Affiliations:** 1 Center for Genomic Regulation, The Barcelona Institute for Science and Technology, 08003 Barcelona, Spain;; 2 Department of Experimental and Health Sciences, University Pompeu Fabra, 08003 Barcelona, Spain; 3 Biomedical Research Networking Center for Rare Diseases (CIBERER), 08003 Barcelona, Spain

## Abstract

Down syndrome (DS) stands as the prevalent genetic cause of intellectual disability, yet comprehensive understanding of its cellular and molecular underpinnings remains limited. In this study, we explore the cellular landscape of the hippocampus in a DS mouse model through single-nuclei transcriptional profiling. Our findings demonstrate that trisomy manifests as a highly specific modification of the transcriptome within distinct cell types. Remarkably, we observed a significant shift in the transcriptomic profile of granule cells in the dentate gyrus (DG) associated with trisomy. We identified the downregulation of a specific small nucleolar RNA host gene, *Snhg11*, as the primary driver behind this observed shift in the trisomic DG. Notably, reduced levels of *Snhg11* in this region were also observed in a distinct DS mouse model, the Dp(16)1Yey, as well as in human postmortem tissue, indicating its relevance in Down syndrome. To elucidate the function of this long non-coding RNA (lncRNA), we knocked down *Snhg11* in the DG of wild-type mice. Intriguingly, this intervention alone was sufficient to impair synaptic plasticity and adult neurogenesis, resembling the cognitive phenotypes associated with trisomy in the hippocampus. Our study uncovers the functional role of *Snhg11* in the DG and underscores the significance of this lncRNA in intellectual disability. Furthermore, our findings highlight the importance of the DG in the memory deficits observed in Down syndrome.

## Introduction

Down syndrome (DS) is caused by trisomy of Homo sapiens chromosome 21 (HSA21) and is the most common cause of genetic intellectual disability, affecting more than 5 million people globally. DS alters central nervous system development and function, impairing cognition, and adaptive behavior. Deficits in hippocampal-mediated learning and memory processes are hallmarks of DS [1, 2], and molecular and cellular defects have been detected in post-mortem fetal DS hippocampus [3, 4]. DS is a disorder of gene expression deregulation, as the triplication of HSA21 results in a global disturbance of the transcriptome that is proposed to contribute to the phenotypic manifestations of DS [5]. This global gene expression deregulation is likely caused by alterations intrinsic to the extra copy of HSA21, such as the overexpression of genes involved in epigenetic regulation. In fact, several studies have suggested chromatin dysfunction in DS [6–11]. However, other possible mechanisms associated with the regulation of chromatin function are still unexplored. Despite HSA21 being the smallest autosome, it is highly enriched in long-non coding RNAs (lncRNAs) [12], which are transcripts with a length of more than 200 nucleotides that are not translated into functional proteins. Moreover, a high number of lncRNAs are abnormally expressed in DS [13–15]. Interestingly, a growing body of evidence from recent studies emphasize the role of lncRNAs in brain function, including learning and memory [16–18] and adult neurogenesis [19, 20], but little is known about their direct function.

Epigenetic mechanisms, including the expression of lncRNAs, are highly cell-type specific, thereby providing a layer of regulation for precise transcriptional control in each cell type. Therefore, their deregulation is expected to have a differential impact on the transcriptome of each cellular subtype. In fact, although bulk RNA-sequencing studies have provided evidence of global disturbance of the transcriptome in the trisomic brain [5, 12, 21], the high cell heterogeneity of the brain tissue greatly hampers the capacity of these studies to elucidate the full complexity of gene expression deregulation in the trisomic brain and to identify specific genes responsible for specific clinical phenotypes.

Here, we used single nucleus RNA sequencing to dissect transcriptional dysregulation associated with specific cell types in the DS mouse model Ts65Dn. Of the DS mouse models generated to date only Ts65Dn, Tc1, Ts66Yah and TcMAC21 are true aneuploid models with a freely segregating supernumerary chromosome, which may be important for some of the DS features not found in other DS mouse models with an intrachromosomal segmental duplication. We selected for our study the Ts65Dn, as it recapitulates many of the features found in DS. However, Ts65Dn mice also carry a triplication of 43 coding genes which are non-orthologous to HSA21, and are not triplicated in human DS. As such, we validated some of our results in a second mouse model of DS the Dp(16)1Yey (Dp16), which has a duplication of the HSA21 orthologous region on MMU16 [22] and also in human postmortem dentate gyrus of DS brains.

Our study revealed a cell-type specific alteration of the transcriptome and detected previously unknown differentially expressed genes in specific neuronal populations. Strikingly, we identified *Snhg11*, a lncRNA, to be specifically downregulated in the trisomic dentate gyrus and we provide evidence for its involvement in neuronal function, adult neurogenesis and hippocampal-dependent memory.

## Results

### Unbiased identification of neuronal subtypes in hippocampus

**Figure 1:**
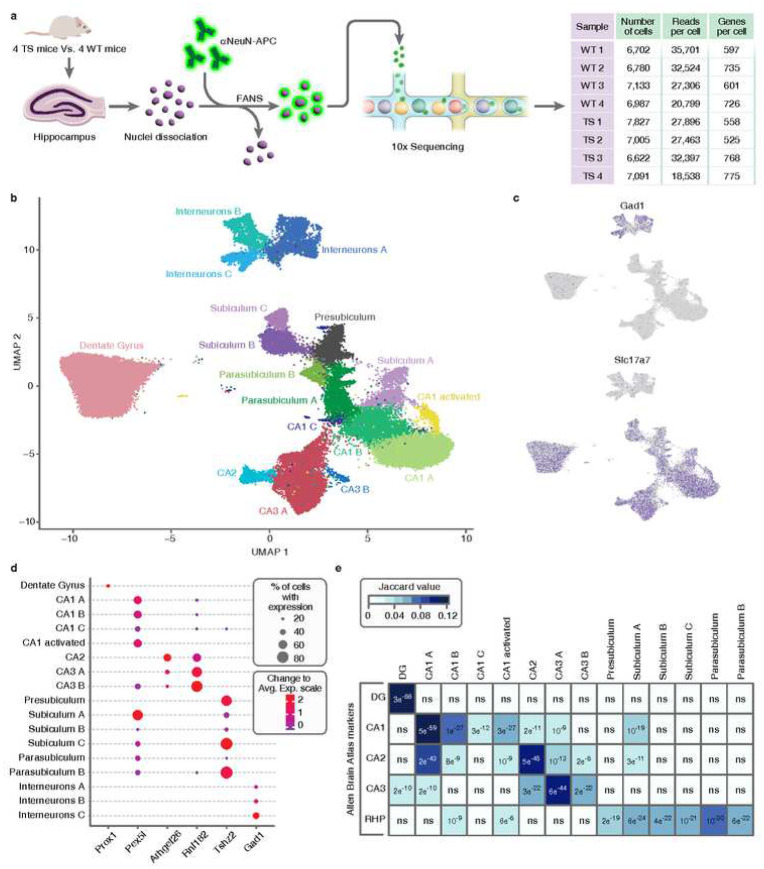
Unbiased identification of neuronal subtypes in hippocampus. a. Work flow of sample preparation depicting isolation of NeuN-positive cells and sequencing results obtained in four wild type (WT) and four trisomic (TS) mice. b. UMAP embedding of all WT and TS hippocampal single nuclei transcriptional pro les showing cell clusters. Cells are colored by cell type. c. Mapping of known cell markers of excitatory (Slc17a7) and inhibitory (Gad1) neurons. d. Dotplot showing enrichment of region-specific markers in each cluster. e. Jaccard overlap coefficient between cluster markers and genes enriched in each hippocampal subregion according to the Allen Brain Atlas. p-values are indicated. FANS uorescent activated nuclear sorting, DG dentate gyrus, RHP retrohippocampal region.

To address the question of how the different neuronal populations are affected in the trisomic (TS) hippocampus, we isolated the NeuN+ population by fluorescence activated nuclear sorting (FANS; see Methods). After FANS, using 10X we sequenced 27602 and 28545 nuclei with high integrity from four WT and four TS mice, respectively (Fig. 1a). Since single nuclei RNA-seq (snRNA-seq) profiles nuclear RNA, our gene expression profile data reflect nascent transcription, as well as the cellular transcriptome. Single-cell feature-barcode matrices were used to embed cells in a K-nearest neighbor graph that defines cell clusters in an unbiased manner. Nuclear transcriptomes were visualized using a uniform manifold approximation and projection (UMAP) plot (Fig. 1b). We detected 17 clusters of cells sharing similar gene expression patterns. These clusters did not result from technical or batch effects (Supplementary Fig. 1). To determine the identity of each cluster, we identified a total of 1191 cluster marker genes for the different hippocampal neuron subpopulations (Supplementary Table 1). Classical gene markers, such as *Slc17a7* for excitatory neurons and *Gad1* for interneurons showed a clear enrichment in the corresponding clusters (Fig. 1c). Glutamatergic cells were further mapped to five hippocampal subregions, by analyzing the expression pattern of the top subregional marker genes (Fig. 1d) of the Allen Brain Atlas [23] (Supplementary Fig. 2a). Examples of those are *Prox1* for the Dentate Gyrus (DG), *Pex5l* for CA1 or *Arhgef26* for CA2 among others. To validate this initial mapping in a systematic manner, we studied the overlap between the top 300 marker genes of each of our clusters and the 300 most differentially expressed genes in each hippocampal subregion identified by the Allen Brain Atlas [23] (Fig. 1e, Supplementary Table 2). Using this same approach, we were also able to identify the major subregions of the RHP, namely presubiculum, subiculum and parasubiculum (Supplementary Fig. 2b).

### Differential expression analysis reveals neuronal subtype-specific alterations in TS hippocampi

**Figure 2:**
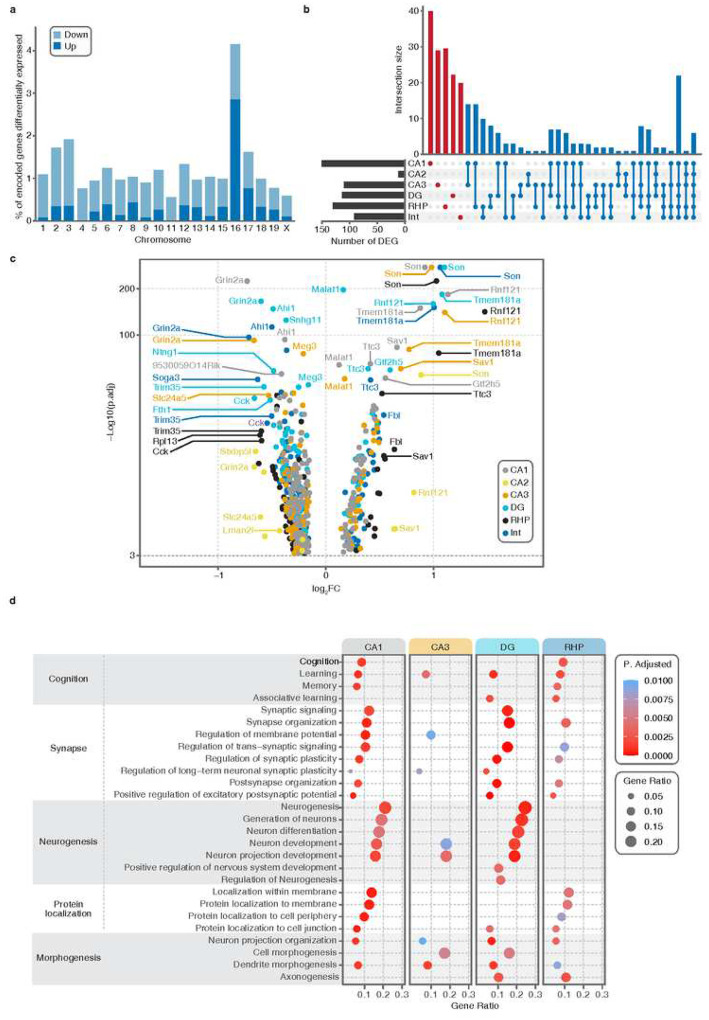
Differentially expressed genes (DEGs) in the trisomic hippocampal cell types. a. Proportion of genes located in each chromosome that were found differentially expressed in the TS hippocampus b. UpSet plot showing the overlap of genes of detected DEGs accross different cell types. DEGs unique to a cell subtype are indicated in red and those shared between 2 or more cell types are indicated by blue dots. The histogram above each plot indicates the number of DEGs for each cell type. The barplots on the left show the total number of DEGs per cellular type and subtype. c. Volcano plot of DEGs colored by each major neuronal type and subtype. d. Biological pathways enriched for DEGs identified in each major hippocampal region. Gene Ratio: proportion of DEGs involved in each category. DEGs differentially expressed genes, DG dentate gyrus, RHP retrohippocampal region, Int interneurons

The gene expression analyses of the trisomic and euploid major neuronal subtypes (CA1, CA2, CA3, DG, RHP and interneurons) revealed a total of 291 differentially expressed genes (DEGs; Supplementary Table 3). The highest portion of DEGs, mostly upregulated, were located to the mouse chromosome 16 (Mmu16) (Fig. 2a), a portion of which is triplicated in Ts65Dn. Among the 42 Mmu17 genes that are triplicated in Ts65Dn but are non-orthologous to HSA21 genes, 7 of them were deregulated. We found that the impact of the trisomy on the transcriptome is cell type specific (Fig. 2b, Supplementary Fig. 3 and Supplementary Fig. 4a). We also found common DEGs in more than one neuronal subtype. The highest number of shared DEGs between two cell types was found between CA1 and CA3, reflecting their close biological identity and functionality. As expected, a decreasing number of genes was found to be commonly deregulated, being the trisomic genes more prone to be overexpressed across cell subtypes (Supplementary Fig. 4a). The cell type specific impact of the trisomy is also illustrated by the finding that most of the DEGs identified at the single nuclei level were not detected in the pseudobulk analysis (i.e. pooling the gene expression values of individual cells, and then calculating the average expression levels of each gene across all cells) of the same samples (Supplementary Fig. 4b).

The identification of cell subtype specific DEGs resulting from the trisomy might be of interest to elucidate the mechanisms that lead to DS hippocampal dysfunction (Fig. 2c, d). For instance, *Epha6*, a key regulator of neuronal and spine morphology, was specifically downregulated in CA1 trisomic neurons. Instead, *Eid1*, which has been associated with an impaired synaptic plasticity of CA1 pyramidal neurons [24], is upregulated in this region. Interestingly, in the DG we found altered expression of genes related to neurogenesis (Fig. 2d), which is severely affected in DS [25–28]. This is the case of the downregulation of *Negr1*, which is a regulator of neurogenesis [29] and the overexpression of *Flrt3*, which is associated with neuronal migration inhibition [30]. Two voltage-gated K+ channel subunits, Kv1.1 and Kv3.1, encoded by *Kcna1* and *Kcnc1*, respectively, were specifically downregulated in interneurons, which could contribute to the known imbalance in excitatory-inhibitory transmission in the trisomic brain [31–34].

Not all trisomic genes were equally altered in each cellular subtype. Regarding the HSA21 orthologous genes, fifteen of the top DS trisomic genes with direct dosage effects reported in two meta-analysis [5, 35] are DEGs in our analysis. Numerous genes on HSA21 were differentially expressed in multiple cell types, such as *Kcnj6*, *Itsn1*, *Synj*, or the (Na+)/myoinositol cotransporter gene (*Slc5a3* [36]) and *Son* [37] being consistently overexpressed in multiple cell types. *Ttc3* [38] and *App* were upregulated in both excitatory and inhibitory neuronal populations whereas *Gart*, one of the top HSA21 genes identified in a meta-analysis [35], is uniquely overexpressed in trisomic CA3 neurons. The expression of important DS candidate genes, such as the dual-specificity tyrosine-(Y)-phosphorylation regulated kinase 1a (*Dyrk1a*), a well-studied gene in DS individuals and mouse models, was not modified in our study, similarly to previous studies [5]. Regarding the expression of genes from the triplicated centromeric part of the Mmu17, we only found seven significantly upregulated. All downregulated genes were non-trisomic. Among them, we found *Grin2a*, encoding the Glutamate Ionotropic Receptor NMDA Type Subunit 2A, to be dramatically reduced in the whole trisomic hippocampus regardless of cellular subtypes.

### The trisomy leads to a major shift in the dentate gyrus transcriptomic profile

**Figure 3:**
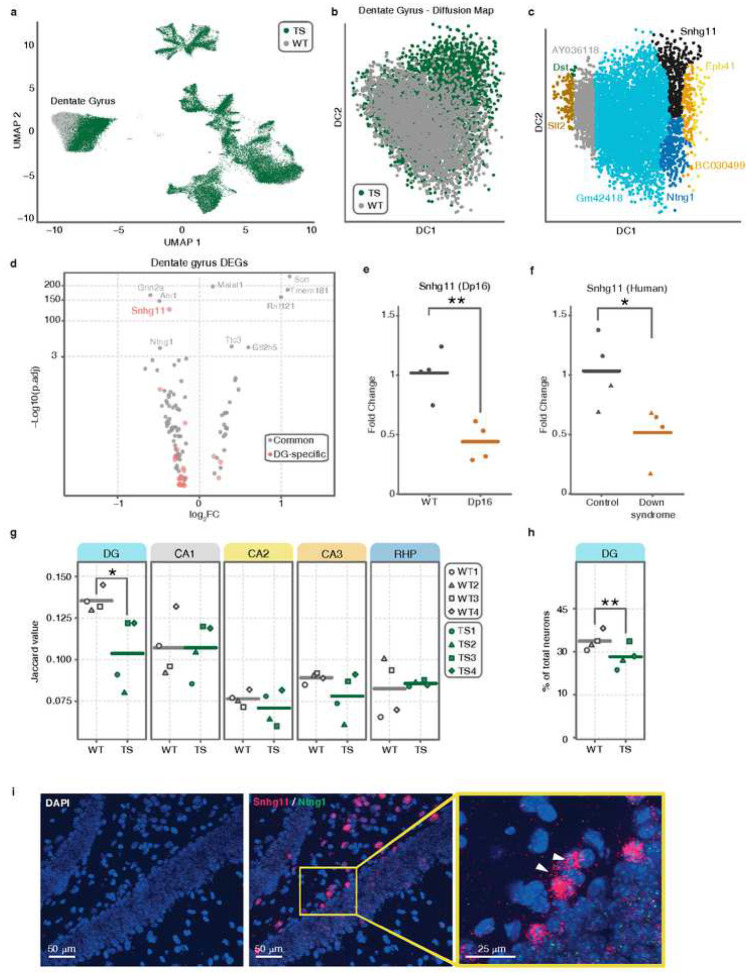
Transcriptomic shift specific to the dentate gyrus. a. UMAP plot of WT (gray) and trisomic (Ts65Dn, TS, green) cells. b. Representation of the two-dimensional embedding of WT and trisomic granule cells by Diffusion map. c. Local gene relevance in the diffusion space showing Snhg11 and Ntng1 local relevance. d. Volcano plot of DG DEGs. Red indicating that they are specifically deregulated in DG. e. RT-qPCR analysis of Snhg11 expression in the DG of WT (gray) and Dp16 (orange) animals. N = 4 male mice per group. ** p < 0.01; Student’s t-test. f. RT-qPCR analysis of Snhg11 expression in the human DG of unaffected controls (gray) and Down syndrome (orange) patients. N = 2 male and 2 female individuals per group. Triangles indicate males and circles indicate females * p < 0.05; permutation test. g. Jaccard values of the overlap between the cluster markers of each WT (gray) and TS (green) sample and the 300 most differentially expressed genes in each hippocampal subregion identified in the Allen Brain Atlas. The bars indicate average values. * p < 0.05; One-way ANOVA followed by TukeyHSD. h. Dotplot showing the percentage of WT (gray) and TS (green) cells belonging to the DG. Each symbol represents an individual (TS1–4; WT1–4).The bars indicate average values. ** p < 0.01; Generalized linear model. i. Representative images of RNAscope in situ hybridization of Snhg11 and Ntng1 in the DG. Each individual 1–2μm dot, represents an individual RNA molecule and its location within cells. Arrow: cytoplasmic localization of Snhg11. Arrow head: nuclear localization of Snhg11

To visualize cell-type specific changes in the transcriptomic profiles of WT and trisomic neurons, we overlapped the UMAP plot from each genotype (Fig. 3a). Strikingly, we observed a major shift in the two dimensional embedding of trisomic granule cells from the trisomic DG compared to WT, whereas no shift was detected in other neuronal subtypes. The trisomic DG shift was quantified by testing if the euclidean distance, a measure of the difference between the gene expression profiles of cells of the two genotypes, was larger than expected by chance, confirming the observed shift in the DG and revealing a milder shift in CA1 (Supplementary Fig. 5 a).

To validate this transcriptomic shift, we subsetted the DG nuclei and repeated the clustering and the two-dimensional reduction by an independent approach, the Diffusion Map, an approach that allows to identify which genes are contributing the most to the position of each cell in the embedding [39, 40]. As in UMAP, we also observed a shift that is specific to the trisomic DG (Fig. 3b), while other subregions and cell subtypes showed a high overlap between genotypes (see examples of CA3 and interneurons in Supplementary Fig. 5b). When further investigating this shift, we identified the small nucleolar RNA host gene 11, *Snhg11* as the gene with the highest global and local gene relevance [39] in the DG diffusion map (Fig. 3c), which indicates that this gene is the main driver of the shift in the two-dimensional embedding, as global gene relevance identifies drivers of the overall embedding, and local gene relevance identifies those of a defined sub-region. *Snhg11* is a member of the non-coding small nucleolar RNA host gene (SNHGs) family. Interestingly, this long non-coding gene is differentially expressed specifically in the trisomic DG, where its expression is strongly reduced (Fig. 3d). The reduced expression of this gene was also observed in the DG of an independent mouse model, the Dp16 (Fig. 3e) and, most importantly, in the DG from DS patients (Fig. 3f and Supplementary Fig. 5c) compared to unaffected controls. Netrin G1, *Ntng1* is also dramatically downregulated in this subregion (Fig. 2c, Fig. 3d) and also shows a high global gene relevance in the two dimensional embedding shift of the trisomic DG (Fig. 3c). *Ntng1* is a known marker of mature granule cells [41], which suggested that cellular and functional identity of trisomic granule cells could be compromised. In fact, we observed a significant loss of DG marker genes, as shown by the significantly lower Jaccard index (Fig. 3g) and a reduced number of neurons in the trisomic DG (Fig. 3h). This reduction of marker identity was specific to the DG, suggesting that the identity of granule cells is compromised. Furthermore, while no compositional changes are observed in the rest of hippocampal subregions, except for a significant increase in interneurons, a finding that is in line with previous studies in DS [31–34], we found the granule cell population to be reduced in the trisomic hippocampus (Fig. 3h, Supplementary Fig. 6) This observation could have a great impact on hippocampus-dependent memory function [42–44].

### ASO-mediated knockdown of *Snhg11 in vivo* leads to trisomic-like transcriptomic alterations

We then addressed the potential involvement of Snhg11 in the DG, with its expression and function in the brain still largely unexplored. As we observed the specific downregulation of *Snhg11* in trisomic mice within the DG, we directed our attention towards this particular subregion within the WT hippocampus. Our analysis involved examining the expression of *Snhg11* and *Ntng1* expression, using in situ hybridization. As previously reported [41], *Ntng1* signal appeared distributed along the granule cell layer as punctuated and with a cytoplasmic localization (Fig. 3f). Similar to most SNHGs [45], we found *Snhg11* expression both in the nucleus and in the cytoplasm. Interestingly, although *Snhg11* signal is evenly distributed in the granule cell layer, cells localized to the subgranular layer displayed a particularly high expression of *Snhg11*, suggesting a potential role of this gene on adult neurogenesis.

To further investigate the function of *Snhg11* in the DG we knocked down its expression in the DG of WT animals. To this aim, we designed three different antisense oligonucleotide (ASO) sequences targeting the conserved exon 5 (Supplementary Table 4 and Supplementary Fig. 7a) of *Snhg11* to trigger its RNAse-H-dependent degradation. To evaluate the efficacy of the ASOs, we transfected the murine neuroblastoma cell line Neuro 2a (N2a) with each ASO targeting *Snhg11* (Snhg11-ASO1–3) or with a negative control (Control-ASO) consisting in a gapmer of the same length as the Snhg11-ASO with a scrambled sequence not targeting any gene. After transfection, we observed different degrees of reduction of *Snhg11* expression as measured by RT-qPCR in cells transfected with each Snhg11-ASOs (Supplementary Fig. 7b). In line with results in different cancer lines [46–50], the ASO-mediated knockdown of *Snhg11* dramatically reduced the proliferation of N2a cells 24h after transfection. Their proliferative capacity was arrested for at least 72h (Supplementary Fig. 7c, d). Among the three ASOs tested, Snhg11-ASO 1 achieved the greatest reduction of *Snhg11* and had the biggest impact on N2a proliferative capacity. For this reason, Snhg11-ASO 1 was selected for subsequent experiments (hereafter referred to as Snhg11-ASO). The impact of Snhg11-ASO was also reproduced in a Colony Formation Assay (CFA), showing that cells transfected with Snhg11-ASO completely lose their capability to grow into a colony (Supplementary Fig. 7e, f).

**Figure 4:**
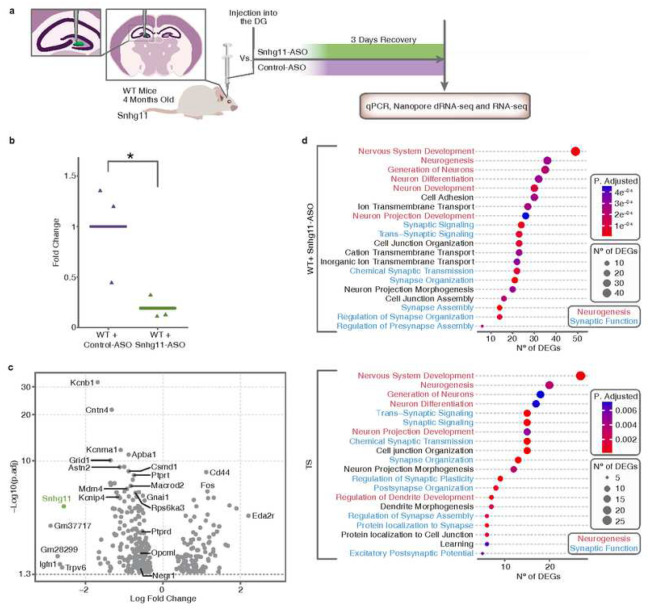
Snhg11 knockdown in the WT Dentate gyrus leads to transcriptomic alterations similar to those of TS mice. a. Outline of the experimental plan. b. Quantitative Real-time PCR (RT-qPCR) analysis of Snhg11 expression in the dorsal hippocampus of injected animals. On the boxplots the horizontal line indicates the median, the box indicates the first to third quartile of expression and whiskers indicate 1.5 × the interquartile range. N = 3 male mice per group. * p<0.05; permutation test. c. Volcano plot for DEGs in the Snhg11 knockdown hippocampus with p.adjusted < 0.05. Snhg11 is colored in green. d. Top biological pathways enriched for DEGs identified in dorsal hippocampi injected with Snhg11-ASO (top) and Ts65Dn DG (bottom). DG dentate gyrus, DEGs differentially expressed genes.

Once the efficacy of the ASO-mediated knockdown of *Snhg11* was confirmed *in vitro*, we investigated its impact *in vivo* by a bilateral injection of 50 μg of either Snhg11-ASO or Control-ASO specifically in each of the hemispheres of the dorsal DG of 4 months-old WT mice (Fig. 4a). The specific knockdown of *Snhg11* in the dorsal hippocampus was confirmed by RT-qPCR (Fig. 4b). To discard possible off-target effects, the expression of *Snhg11* was also measured in the ventral hippocampus (Supplementary Fig. 8a). We also observed a reduced expression of the two small nucleolar RNAs (snoRNAs) hosted by the gene (Supplementary Fig. 8b and c).

In order to investigate the role of these snoRNAs (Gm25187 and Gm25129) in the *Snhg11* knockdown samples, we performed direct RNA nanopore sequencing (DRS) in Control-ASO and Snhg11-ASO injected samples, in biological duplicates. This technique can identify and quantify changes in ribosomal RNA modification levels by measuring the difference in base-calling errors (e.g. mismatches) between two samples [51]. Analysis of the DRS data revealed no significant changes in 5S, 5.8S and 28S transcripts between Control-ASO and Snhg11-ASO samples. By contrast, it showed modest changes at two known pseudouridine (pU) positions of the 18S rRNA (Supplementary Fig. 8d). More specifically, we observed a ~5% decrease in mismatch frequency at pU 18S:407, which is a known target of snoRNA 71 (Gm25187), in one of the two replicates (Supplementary Fig. 8e and f). Notably, the replicate that did not show significant changes at 18S:407 showed a reduced efficiency of the snoRNA 71 knockdown (Supplementary Fig. 8c; R1) compared to the other replicate (Supplementary Fig. 8c; R2). In addition, we also found a reduction in the pU modification levels of 18S:1137 (~5% loss in mismatch frequency in both replicates (Supplementary Fig. 8e)), which we speculate to be a target of ACA60 (Gm25129; Supplementary Fig. 8f). This analysis suggests that while minor differences in the rRNA modification levels are observed in the rRNA upon *Snhg11* knockdown, it is unlikely that these extremely modest differences are responsible for the phenotypes observed.

We also tested whether the reduction in *Snhg11* expression was accompanied by a loss of granule cell identity and found a reduction in *Ntng1* expression (Supplementary Fig. 8g), similarly to what we observed in the TS DG.

In order to investigate the role of *Snhg11* in the DG and its contribution to the detected DG-dependent transcriptional changes in Ts65Dn, we performed RNA-sequencing on the dorsal hippocampus of animals injected either with Snhg11-ASO or Control-ASO. Strikingly, the reduction of *Snhg11* led to an important transcriptional alteration, with 337 genes being differentially expressed (Fig. 4c, Supplementary Fig. 9a and Supplementary Table 5). Interestingly, despite the technical differences between both approaches, there is a significant overlap between these deregulated genes and deregulated genes found in the trisomic DG in the snRNA-seq experiment (Supplementary Fig. 9b). Most of these genes (11 out of 13) were downregulated and their deregulation showed a robust correlation between both experiments (Supplementary Fig. 9c). These included genes that are important for neurogenesis, such as *Negr1* [29] and *Ptprd* [52], and for synaptic function, such as Dpp6 [53]. In line with this observation, the Gene Ontology analysis of the downregulated genes revealed that the pathways affected upon *Snhg11* knockdown are mainly related to neurogenic and synaptic function categories (Fig. 4d), a similar enrichment that was observed among genes downregulated in the trisomic DG (Fig. 4d). Altogether, our results support the relevance of *Snhg11* for DG-dependent deficits in DS.

### *Snhg11* knockdown recapitulates DS DG-dependent neurogenesis, synaptic plasticity and memory deficits

**Figure 5:**
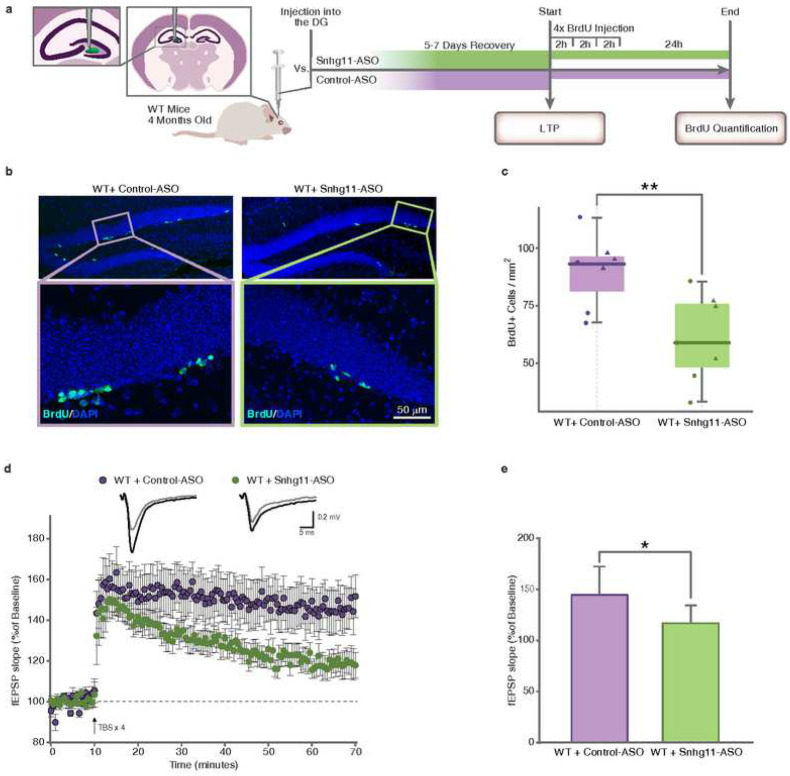
Snhg11 knockdown in the WT dentate gyrus leads to deficits in neurogenesis and long-term potentiation. a. Outline of the experimental plan. b. Representative images of BrdU labeled cells in sections of the DG in a Control-ASO-injected mouse (left) and Snhg11-ASO-injected mouse (right). c. Quantification of BrdU+ cells in the DG. On the boxplots, the horizontal line indicates the median, the box indicates the first to third quartile of expression and whiskers indicate 1.5 × the interquartile range. N = 3 male mice and 4 female mice per group. Triangles indicate males and circles indicate females. ** p< 0.001; Two-way factorial ANOVA followed byTukeyHSD test. d. Time course of mean fEPSPs slope in basal condition and after LTP induction with four Theta Burst Stimulation protocol in DG of WT Control-ASO-injected mice (purple; n (slices) = 7, N (mice) = 4; 146.06 27.79%) and Snhg11-ASO-injected mouse (green; n (slices) = 10, N (mice) = 5; 118.45 17.34%). Data were normalized for each slice with respect to the average baseline slope recorded during 10 minutes and recordedduring 60 minutes after LTP induction. e. Average fEPSP slope 50 minutes after TBS protocol. Data are represented as mean +/− SEM. * p< 0.05; Student t-test

To further investigate the relationship between *Snhg11* and adult neurogenesis, we quantified the generation of new neurons in the DG of animals injected either with Snhg11-ASO or with Control-ASO (Fig. 5a). To this aim, we sacrificed the animals 3 days upon injection and processed their brains for histology. In our experiments, the density of BrdU^+^ cells in the DG region was reduced by ~30% in Snhg11-ASO injected mice compared to Control-ASO injected mice (Fig. 5b, c). In line with the observation of an altered transcriptome affecting neurogenic pathways upon *Snhg11* knockdown, these results indicate that *Snhg11* is required for optimal adult neurogenesis in the DG.

In order to determine the role of *Snhg11* in DG synaptic plasticity we induced long-term potentiation (LTP) in mouse brain slices. We stimulated the perforant pathway (PP) and recorded field excitatory postsynaptic potentials (fEPSPs) in the molecular layer of the DG. First, we performed an Input/Output (I/O) curve in order to analyze differences in basal synaptic transmission, and we did not detect differences between Control-ASO and Snhg11-ASO (Supplementary Fig. 10). LTP was induced with a theta burst stimulation (TBS) protocol in Control-ASO and Snhg11-ASO injected mice. In our experiments, 5 to 7 days after the injection, Snhg11-ASO knockdown mice showed a significant deficit in hippocampal LTP, indicating a potential role of *Snhg11* in synaptic plasticity processes (Fig. 5d, e). The affectation of some genes related with normal synaptic function in the transcriptome of *Snhg11* knockdown supports the idea that *Snhg11* has a crucial role in synapse plasticity.

**Figure 6.**
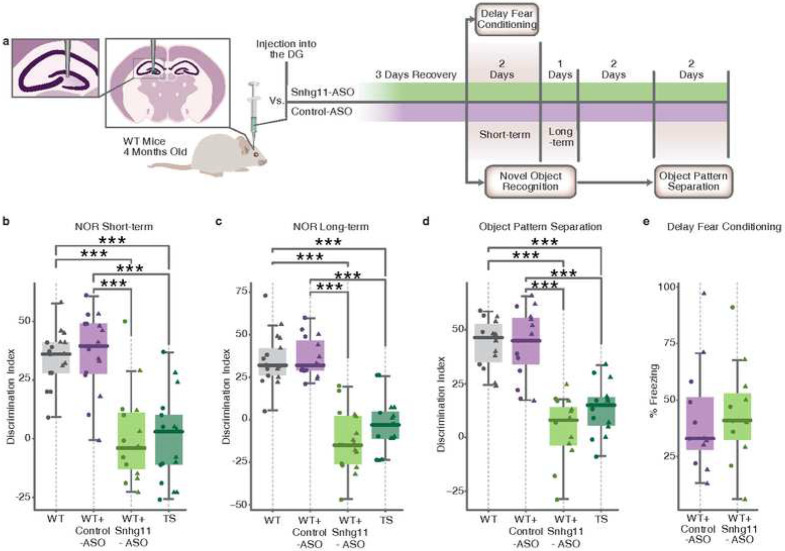
Snhg11 knockdown effects on hippocampal-dependent memory in WT, WT injected either with Control-ASO or Snhg11-ASO and TS mice. a. Outline of the experimental plan. b-c. Novel Object recognition memory after 1 hour (b) and after 24 hours (c). d. Pattern separation memory after 1 hour. e. Delay fear conditioning. Triangles indicate males and circles indicate females. *** p<0.001; Two-way factorial ANOVA followed by TukeyHSD test. DG dentate gyrus, NOR Novel Object Recognition test.

Our results indicated that *Snhg11* plays an important function in adult neurogenesis and synaptic plasticity in the DG, which are fundamental processes for hippocampal-dependent memory. To assess whether the observed alterations upon *Snhg11* knockdown lead to memory deficits that recapitulate the ones observed in TS animals, we performed a behavioral battery in male and female WT animals injected either with Snhg11-ASO or Control-ASO injection (Fig. 6a) and also in non-injected WT and TS animals. We started the behavioural battery 3 days after the injection of the ASOs.

Strikingly, the reduction of *Snhg11* had a dramatic impact on all the cortico-hippocampal memory paradigms tested. In the Novel Object Recognition (NOR) task, which measures recognition memory, non injected and WT mice injected with the Control-ASO properly acquired memory and recalled it both after 1 hour (Fig. 6b) or after 24 hours (Fig. 6c) as shown by the pronounced preference for the novel object measured by the Discrimination Index (DI). Instead, WT animals that were administered with Snhg11-ASO failed to display object recognition both at short or long term to a similar extent as trisomic mice (Fig. 6b, c). In order to address more specifically the impact of *Snhg11* knockdown on DG-dependent tasks, we assessed whether Snhg11-ASO-injected animals presented an altered spatial pattern separation memory, which strictly depends on the DG [54, 55]. For this, we used a paradigm known as Object Pattern Separation [56]. In this case, the loss of *Snhg11* led to an even more dramatic reduction of memory performance than the one observed in TS, whereas both WT and Control-ASO-injected mice showed a high DI (Fig. 6d).

In order to confirm the DG-specific impact of the Snhg11 knockdown, we lastly subjected a separate group of animals to the Delay Fear Conditioning paradigm, which is a memory task known to be independent of hippocampal function (Fig. 6e). As expected, in this case we did not observe any performance differences between Control-ASO and Snhg11-ASO-injected animals. Similarly, no changes were observed on motor activity, explorative behavior or anxiety (Supplementary Fig. 11).

## Discussion

DS is a disorder of gene expression deregulation, as the triplication of HSA21 results in a global disturbance of the transcriptome that is proposed to contribute to its phenotypic manifestations. However, the mechanisms regulating gene expression are highly cell-type specific, and thus, the full complexity of gene deregulation in DS may be missed in bulk studies. This is particularly relevant in those tissues with high cellular heterogeneity such as the hippocampus, where the extent to which each cellular subtype is affected by the trisomy remains elusive.

To overcome this limitation, we have generated the first single-nuclei atlas of a trisomic hippocampus by characterizing the transcriptome of tens of thousands of individual hippocampal neurons in parallel in the Ts65Dn mouse model of DS. Notwithstanding the fact that Ts65Dn mice carry a triplication of 43 coding genes which are non-orthologous to HSA21, and are not triplicated in human DS, we chose to perform our snRNAseq on this DS model as Ts65Dn is a true aneuploid model with an extra freely segregating marker chromosome, which may be important for some of the DS features. A new model, TcMAC21 mouse model, has been recently generated, that overcomes the drawbacks of previous models in that it is not mosaic and contains a near complete (93%) HSA21, but it was not available when we started the project. Furthermore, there is uncertainty regarding the interactions between proteins encoded by human genes and mouse orthologs [57, 58], and the presence of a significant number of non-coding human genes (>400) with uncertain effects on the mouse transcriptome.

In the present study we show that, although it has been previously suggested that aneuploidies induce a uniform transcriptional deregulation in different cell lines [45, 46], the differential expression analysis of each cellular subtype revealed that almost half of the total DEGs were uniquely deregulated in a specific neuronal subtype. Thus, our results indicate that the impact of the trisomy on the transcriptome is dependent on the cellular identity, suggesting cell type-specific epigenetic mechanisms. In fact, besides the expected upregulation of certain triplicated genes known to be critical for DS hippocampal deficits such as *App* [47, 48] and *Synj1* [49], we have also identified relevant genes that are uniquely deregulated in specific populations. This is the case of *Kcna1* and *Kcnn1*, two genes encoding voltage-gated K+ channel subunits, which are specifically downregulated in the interneuron population, potentially contributing to the excitatory-inhibitory imbalance in the DS brain. In this regard, we also observed an increased inhibitory population in the trisomic hippocampus, in line with previous studies [31–34]. The extent of the cell type-specific impact of the trisomy is also illustrated by the important differences in DE genes found between pyramidal neurons located in different subregions of the CA. As discussed above, one of the limitations of our study is the use of Ts65Dn, which besides overexpressing two thirds of the triplicated HSA21 ortholog genes, it is also also trisomic for non-HSA21 orthologs. Importantly, among the 42 Mmu17 genes that are triplicated in Ts65Dn but are non-orthologous to HSA21 genes, only 7 of them were deregulated.

Although one might think that all trisomic genes should be equally overexpressed in each cellular subtype, this was not the case in our study. Numerous genes on HSA21 were differentially expressed in multiple cell types, including *Kcnj6*, a potassium inwardly rectifying channel, *Itsn1* and *Synj*, involved in synaptic vesicle recycling and/or development of normal synaptic morphology [62–64], or *Slc5a3* the (Na+)/myoinositol cotransporter gene involved in abnormalities in myo-inositol metabolism [36]. The E3 ligase, *Ttc3* and *App*, both involved in Alzheimer’s disease [65–67] were upregulated altered in both excitatory and inhibitory neuronal populations in the Ts65Dn hippocampus. The splicing regulator Son was consistently overexpressed in every cell type examined, whereas *Gart*, one of the top HSA21 genes identified in a meta-analysis [35], was uniquely overexpressed in trisomic CA3 neurons. The expression of dual-specificity tyrosine-(Y)-phosphorylation regulated kinase 1a (*Dyrk1a*), a well-studied triplicated gene in DS individuals and mouse models, was not detected as modified in our study. This result is explained by the low level of expression of the gene in our nuclear RNA dataset, not reaching the minimum threshold to be included in the analyses. However, previous expression profiling studies involving the brain of different trisomic mouse models did also not find *Dyrk1a* differentially expressed [5]. All downregulated genes in our study were non-trisomic. Among them, we found *Grin2a*, encoding the Glutamate Ionotropic Receptor NMDA Type Subunit 2A, to be dramatically reduced in the whole trisomic hippocampus regardless of cellular subtypes. Importantly, the DG was particularly affected. In this region, which is the only neurogenic niche in the adult mouse hippocampus, we found a transcriptional deregulation of genes related to neurogenesis. Although an alteration of neurogenesis has been proposed to be a neurobiological correlate of intellectual disability in DS [68], few studies have focused on this hippocampal subregion. Those have shown that cell proliferation from early postnatal stages is reduced in the subgranular zone of fetuses with DS and of Ts65Dn mice [3, 69]. The number of differentiated neurons is also reduced in individuals with DS [70]. This defective neurogenesis derived from the trisomy continues into adulthood [71, 72]. Here, we found a granule cell hypocellularity in TS mice, which is possibly contributed by the reduced neurogenesis. Importantly, in this region, a relatively small alteration in the number of granule cells might have an impact in learning and memory [42].

Most of the research on the mechanisms leading to an impaired neurogenesis in DS has focused on triplicated genes on HSA21 [50]. Here we also identified specific non-triplicated genes such as *Negr1, Ptprd* and *Flrt3*, the deregulation of which could contribute to the defective neurogenesis in the trisomic DG. Strikingly, the DG also showed a specific shift in the UMAP representation of the nuclei transcriptome. Along with this observation, the trisomic DG showed a significant loss of marker genes defined by the Allen Brain Atlas [23]. In particular, *Ntng1*, a marker of mature granule cells [41], was consistently downregulated in the trisomic DG. The downregulation of this gene has been linked to the immature DG phenotype, which is proposed to contribute to several neuropsychiatric disorders [73–75].

Strikingly, we also detected a marked reduction of the expression of *Snhg11*, a lncRNA involved in cell proliferation in different types of cancer [46–50] which was the main contributor to the transcriptomic shift. In recent years, there has been a growing interest on lncRNAs, which have been shown to act as essential epigenetic regulators [76] and have been functionally and mechanistically linked with neurobiological processes related with neuronal proliferation and differentiation [19, 20] and with learning and memory [16–18], as well as with neurological disorders [77, 78]. However, a conclusive link between lncRNAs and DS pathophysiology has not been reported so far. In our dataset, besides *Snhg11*, we found other 7 lncRNAs differentially expressed, 5 downregulated and 2 upregulated, of which *Meg3* and *Malat1* were deregulated across several neuronal types.

The diffusion map showed that *Snhg11* was the gene with the highest relevance to explain the DG shift and was specifically and very significantly downregulated in this region. For these reasons, we focused our further experiments on this lncRNA. Given the limitations of Ts65Dn, we validated the DG-specific downregulation of *Snhg11* in an independent DS mouse model, the Dp16, which carries all Mmu16 orthologs from HSA21 as a duplication [81] and, most importantly, in the dissected DG from human DS patients. Altogether, this indicates that *Snhg11* deregulation is not limited to Ts65Dn and could constitute a common mechanism in DS. Despite the high expression of *Snhg11* in mouse hippocampus, its role in neuronal function had not been yet explored. Thus, we explored the role of *Snhg11* by knocking down the gene in the DG of WT mice. Although the ASO-mediated reduction was greater than the one observed in the trisomic DG, our results revealed the key role of this lncRNA in the hippocampal function. In particular, the reduction of *Snhg11* resulted in an important transcriptional alteration in the DG, mainly leading to a downregulation of genes related to neurogenic and synaptic function categories, which we also found enriched among the downregulated genes in the trisomic DG, supporting the relevance of *Snhg11* for DG-dependent deficits in DS.

Several mechanisms could be responsible for the wide impact of *Snhg11* on gene expression. The group of lncRNAs to which *Snhg11* belongs, the small nucleolar RNA host genes (SNHGs) class, has wide-range mechanisms of action. In the nucleus, they can modulate gene expression either by influencing DNA methylation through the modulation of methylation enzymes or by interacting with transcription factors repressing gene transcription [45]. We demonstrated that *Snhg11* expression is not confined to the nucleus, but it is also expressed in the cytoplasm, where other members of the SNHG family have been shown to modulate gene expression by miRNA sponging [45], although further evidence is needed to consolidate this mechanism of action [79].

In addition, the small nucleolar RNAs (snoRNAs) hosted by these genes can also have an impact on cell function. *Snhg11* harbours two snoRNAs that are highly conserved between human and mice: *Gm25129* (*ACA60* in humans) and *Gm15187* (*SNORA71* in humans). Both of them are H/ACA box snoRNAs, which are a class of snoRNAs involved in pseudouridylation of rRNA, a key step for ribosome biogenesis [80], which is essential for cell proliferation. In fact, the downregulation of both snoRNAs upon *Snhg11* knockdown results in a small reduction of pseudouridylation levels at two ribosomal 18S positions, although these modest differences are unlikely to be responsible for explaining the phenotypes observed in this work. SnoRNAs are also increasingly regarded as key regulators of gene expression [81–83]. Furthermore, dynamic changes in snoRNA expression have been reported in different brain areas upon fear conditioning, indicating a possible role in learning and memory [84–86]. Therefore, the reduction of *Snhg11* expression in the DG could have far-reaching implications for neuronal function and identity.

As expected from these observations, the *in vivo* knockdown of *Snhg11* had an important functional impact in the WT DG. Previous reports indicated that *Snhg11* is upregulated in various types of cancer [46–50], where it promotes cellular proliferation, mainly through the regulation of the Wnt/β-catenin signaling pathway, which is fundamental for adult neurogenesis [87] and is affected in DS [88]. In line with these results, we observed a significant decrease of the adult neurogenic capacity upon *Snhg11* reduction. However, we did not find any significant deregulation of the Wnt/β-catenin signaling pathway, but other deregulated genes, such as Ptprd [52], *Macrod2* [89] and *Negr1* [29], found downregulated both in TS DG and in *Snhg11* knockdown tissue may account for this phenotype. Nevertheless, this question warrants further investigation.

In order to investigate the functional implications of *Snhg11* knockdown on synaptic plasticity we analyzed LTP in the DG of mice injected with the Snhg11-ASO. LTP, one form of synaptic plasticity, is considered one of the basis of learning and memory processes [90] and is impaired in the DG of TS [91, 92]. Interestingly, we observed a significant decay of LTP in Snhg11-ASO injected mice, demonstrating the role of this lncRNA in synaptic plasticity. The downregulation of key genes for synaptic transmission and plasticity observed upon *Snhg11* knockdown could contribute to this phenotype. Among them, the expression of *Negr1* is found reduced both in Snhg11-ASO-injected animals and in the trisomic DG and its reduction has been linked to LTP deficits [29]. These results, together with the reduced adult neurogenesis, could underlie memory deficits that recapitulate the hippocampus-dependent phenotypes observed in TS animals.

In line with these results, the reduction of *Snhg11* expression in the DG led to dramatic hippocampal-dependent memory deficits that recapitulate those observed in TS animals. Upon *Snhg11* knockdown, we detected a strong impairment of both long-term and short-term recognition memory in Snhg11-ASO injected mice, that is similar to the one observed in TS animals, indicating that the hippocampal function is compromised in these animals. Importantly, we also observed a striking impairment of the pattern separation function, which is unique to the DG [54, 55], showing that the DG is specifically affected. Although the specific mechanisms remain to be explored, the long and short-term memory deficits could be explained by the defective synaptic plasticity resulting from the reduction of *Snhg11* expression. It has been shown that newly generated neurons are also important for long-term memories and particularly for pattern separation [44, 93, 94]. However, the link between the described phenotypes and the observed deficits in adult neurogenesis is uncertain, as the behavioral tests were conducted between 1 and 2 weeks after the Snhg11-ASO injection, while newborn granule cells take around 2 weeks to form stable functional synapses and be integrated in the hippocampal circuit [95]. Nevertheless, given the transcriptomic alteration of genes involved in neuronal differentiation and survival, it is also possible that the knockdown of *Snhg11* interferes with the integration of neurons born previously to the injection of the ASO, thereby contributing to the DG-dependent memory deficits.

In conclusion, our results provide evidence of the zcell-type specificity of the transcriptomic alterations in DS and constitute a high-resolution gene expression atlas of the complex population of neurons that integrates the hippocampus both in WT and in a mouse model of DS. We believe that the identification of *Snhg11* as a key player in the DG constitutes an important contribution to support the involvement of lncRNAs in neuronal function and in neurodevelopmental disorders such as DS, highlighting their functional importance in synaptic plasticity, adult neurogenesis and cognitive function. Given that lncRNAs represent a significant population of the human transcriptome, further research is warranted to investigate their role in DS.

## Methods

### Animals

We used two different strains for our experiments: for the in vivo experiments we used Ts65Dn, which contains two thirds of genes orthologous to HSA21, and their wild type littermates, which were also used for Snhg11 ASO injections. For in vivo validation experiments, dissected dentate gyri from 4 month-old male Dp(16)1Yey/+ mutant strain that contains one copy of mouse MMU16 with the targeted sequence between, and including, the lipase, member I (Lipi) gene and the zinc finger protein 295 (Zfp295) gene were kindly provided by Dr. Marie Claude Potier (ICM, France). For the Ts65Dn, experimental mice were generated by crossing of Ts65Dn females to C57/6Ei × C3H/HeSnJ F1 hybrid (B6EiC3) males. The parental generation was obtained from the research colony at the Jackson Laboratory (B6EiC3Sn.BLiA-Ts(1716)65Dn/DnJ; Stock No: 005252). Genotypes of mice were authenticated by PCR assays on mouse tail samples with an in-house protocol. Mice were housed in standard cages (156 × 369 × 132 mm), with food and water available *ad libitum* in standard conditions (12:12 light cycle; 400 lux). Sawdust and nesting materials in each cage were changed once a week, but never on the day before or the day of testing, to minimize the disruptive and stressful effect of cage cleaning on behavior.

All experiments followed the principle of the “Three Rs”: replacement, reduction and refinement according to Directive 63 / 2010 and its implementation in Member States. The study was conducted according to the guidelines of the local (law 32/2007) and European regulations (2010/63/EU) and the Standards for Use of Laboratory Animals no. A5388–01 (NIH), and approved by the Ethics Committee of Parc de Recerca Biomèdica (Comité Ético de Experimentación Animal del PRBB (CEEA-PRBB); MDS 0035P2). Reporting followed the ARRIVE (Animal Research: Reporting of In Vivo Experiments) guidelines with the modifications suggested by the Trisomy 21 Research Society for work with DS mouse models [96]. The CRG is authorized to work with genetically modified organisms (A/ES/05/I-13 and A/ES/05/14). Experimenters were blinded to the genotype for all experiments. Sample size was determined based on previous single cell and behavioural studies that demonstrated sufficient statistical power.

#### Single Nucleus RNA sequencing

##### Nucleus Isolation

Ts65Dn and wild type mice (N=4 per genotype) were sacrificed by cervical dislocation and the hippocampus of each mouse was dissected and placed in chilled Hanks’ Balanced Salt Solution (Sigma #55021C). The “Frankenstein” protocol [97] was followed to obtain a nuclei suspension. Briefly, each hippocampus was transferred to a new tube containing 500μL chilled EZ Lysis Buffer (Sigma #3408) and homogenized using a sterile RNase-free douncer (Mettler toledo #K749521–1590). The resulting homogenate was filtered using a 70μm strainer mesh to remove remaining chunks of tissue and centrifuged at 500g for 5 minutes at 4°C. The pellet containing the nuclei was resuspended in 1.5mL EZ Lysis Buffer and centrifuged again. The supernatant was removed and 500μL of Nuclei Wash and Resuspension Buffer (NWRB, 1X PBS, 1% BSA and 0.2U/μL RNase inhibitor (Thermo Scientific #N8080119) were added without disturbing the pellet and incubated for 5 minutes. After incubation, 1mL of NWRB was added and the pellet resuspended. The nuclei suspension was centrifuged again and the washing step was repeated with 1.5mL of NWRB. After an additional centrifugation, nuclei were resuspended in 500μL of 1:1000 anti-NeuN antibody conjugated with AlexaFluor 647 (ab190565 Abcam) in PBS and incubated in rotation for 15 minutes at 4°C. After incubation, nuclei were washed with 500μL of NWRB and centrifuged again. Last, nuclei were resuspended in NWRB supplemented with DAPI and filtered with a 35μm cell strainer to obtain a single-nuclei suspension.

##### 10x single-cell barcoding, library preparation and sequencing

Fluorescent activated nuclear sorting (FANS) was used to sort NeuN+ neuronal nuclei. Using a 70μm nozzle to minimize the volume deposited, 10.000 nuclei from each sample are sorted directly into a 96-well plate prefilled with 10X RT buffer prepared without the RT Enzyme Mix. After sorting, the RT Enzyme C is added and the volume of each well topped up to 80ul with nuclease free water. Last, 75uL of the nuclei plus RT mix are loaded into the Chromium Single Cell Chip. All downstream cDNA synthesis), library preparation, and sequencing were carried out as instructed by the manufacturer (10x Genomics Chromium Single Cell Kit Version 3). Libraries were prepared and sequenced in two separate experiments. In the first three wild type and three trisomic hippocampi and in the second one hippocampus per genotype were processed. For each experiment, all libraries were pooled and sequenced on NovaSeq 6000 S1 to an average depth of approximately 20.000 reads per cell.

##### 10X data pre-processing

The resulting reads were aligned to the reference genome and converted to mRNA molecule counts using the Cellranger pipeline (CellRanger v3.0.1 [98]) provided by the manufacturer. For every nucleus, we quantified the number of genes for which at least one read was mapped, and then excluded all nuclei with fewer than 200 or more than 2500 detected genes, to discard low quality nuclei and duplets, respectively. Genes that were detected in fewer than six nuclei were excluded. Expression values Ei,j for gene i in cell j were calculated by dividing UMI counts for gene i by the sum of the UMI counts in nucleus j, to normalize for differences in coverage, and then multiplying by 10,000 to create TP10K (transcript per 10,000) values, and finally computing log2(TP10K + 1) using the NormalizeData function from the Seurat package v.2.3.4 [99].

##### Batch Correction and scaling data matrix

Since samples were processed in two different experiments, batch correction was done using Harmony [100] on the normalized dataset. The batch-corrected data was scaled using the ScaleData function from Seurat [99] with default parameters (v.2.3.4), yielding the relative expression of each gene by scaling and centering. The scaled data matrix was then used for dimensionality reduction and clustering. To rule out the possibility that the resulting clusters were driven by batch or other technical effects, we examined the distribution of samples within each cluster and the distribution of the number of genes detected across clusters (as a measure of nucleus quality). Overall, the nuclei separated into distinct point clouds in t-distributed stochastic neighbor embedding (t-SNE) space that were not driven by batch; each cluster was a mixture of nuclei from all technical and biological replicates.

##### Dimensionality reduction, clustering and visualization.

We used the scaled expression matrix restricted to the variable genes for principal component analysis (PCA), using the RunPCA method in Seurat (a wrapper for the irlba function), computing the top 60 principal components. Scores from these principal components were used as the input to downstream clustering and visualization by Uniform Manifold Approximation and Projection (UMAP).

Clustering was performed using the Seurat functions FindNeighbors and FindClusters (resolution = 0.6). Clusters were then visualized with UMAP. Reference anchors were identified between genotypes before integration with the IntegrateData function, and integrated data were then processed by the same methods.

##### Identification of marker genes of individual cell clusters

Cluster-specific marker genes were identified using the FindAllMarkers function from Seurat [99], utilizing a negative binomial distribution (DESeq2). A marker gene was defined as being >0.25 log-fold higher than the mean expression value in the other clusters, and with a detectable expression in > 20% of all cells from the corresponding cluster both in WT and TS groups. In this way, we were able to select markers that were highly expressed within each cluster, while still being restricted to genes unique to each individual cluster. We evaluated the enrichment of region-specific markers in each cluster by calculating the Jaccard overlap coefficient between cluster markers and genes enriched in each hippocampal subregion according to the Allen Brain Atlas [23, 101]. To find region-enriched genes, we used the “Differential Search” function in the Allen Brain Atlas web, setting the region of interest as “Target Structure” and the whole Hippocampal Formation as the “Contrast Structure”. We set an expression threshold of 2.5.

##### Identification of DEGs between WT and TS

All clusters identified as belonging to the same cell type were merged for differential gene expression analyses. Within each cell type, WT and TS samples were compared for differential gene expression using Seurat’s FindMarkers function. To be included in the analysis, the gene had to be expressed in at least 10% of the cells from one of the two groups for that cell type and there had to be at least a 0.1 fold change in gene expression between genotypes. After correcting for multiple testing, only genes with a p adjusted value < 0.001 were considered for downstream analyses.

##### Gene set enrichment

The differential expression signatures from each cellular subtype were tested for enriched Gene Ontology processes, using a hypergeometric test (shinyGO [102]), and corrected for multiple hypotheses by false discovery rate (FDR). Processes with p adjusted value < 0.05 were reported as significantly enriched. The complete list of genes present in the dataset was used as the universe for the hypergeometric test.

##### Diffusion map

For the automatic identification of relevant genes from low-dimensional embeddings of our single-cell RNA-seq data, we used diffusion maps [40]. The diffusion components were calculated using the cell embedding values in the top 15 principal components (generated on the scaled expression matrix restricted to the most variable genes), using the DiffusionMap function from the destiny package [40] in R (with k = 30 and a local sigma). We then chose the top two diffusion components for data visualization. To investigate the DG-specific shift, we took advantage of the gene_overlap [39] function of the destiny package, that allows us to identify drivers of cell embedding at a global or subregion level from non-linear low-dimensional embeddings such as UMAP or Diffusion Map

##### Cellular proportion analysis

To gain insight into the cell type alterations in the trisomic hippocampus, the relative proportion of the number of nuclei in each cell type was normalized to the total number of nuclei captured from each library. To determine if any changes in cell-type proportion were statistically significant, we implemented single cell differential composition analysis (scDC) [103] to bootstrap proportion estimates for our samples. We employed a linear mixed model (random effect of subject) to determine if any changes in cell-type proportion were present.

##### Quantitative assessment of global transcriptome shifts

The quantification was done as explained in [104]. Briefly, we generated two representative cells, one for the WT group and the other for the TS group. This was done by calculating the average gene expression of each gene for each genotype group within that cell type. We then calculate the Euclidean distance in gene expression between these representative cells as a metric to quantify the effect of the trisomy on each cell type. To determine if the observed Euclidean distance between WT and TS cells within each cell type was significantly larger than that of random cells, we estimated a null distribution by calculating the Euclidean distance between randomly sampled cells of the given cell type. This permutation approach was repeated for a total of 1000 times to generate the null distribution, which is compared to the Euclidean distance generated from the representative WT and TS cells to determine an empirical p value. To correct for multiple testing across all the cell types tested, we applied a Bonferroni correction to retrieve adjusted p values.

##### RNAscope

In situ hybridization was performed using RNAscope^®^ Multiplex Fluorescent assay (V1) (Advanced Cell Diagnostics) probes and reagents. Naive animals were sacrificed and transcardially perfused with 50mL of chilled PBS followed by fixation with 50–100mL of 4% depolymerised paraformaldehyde (PFA). Following hippocampus dissection, tissues were placed in 4% PFA and post-fixed overnight at 4°C. Tissues were then immersed in increasing concentrations of sucrose (10%, 20% and 30%) over three days, increasing the concentration every 24 hours. Once embedded in sucrose, tissues were dried and frozen at −80°C prior to cryosectioning. 14 μm sections were obtained and mounted on Superfrost charged slides and stored at −80°C until being processed. In situ hybridization was performed following the manufacturer’s guidelines. Custom RNAscope^®^ target specific oligonucleotide (ZZ) probes were designed by Advanced Cell Diagnostics targeting nucleotides 2–1604 for *Snhg11* (NR_164123.1) and 1130 – 2109 for *Ntng1* (NM_030699.2).

#### Snhg11 downregulation validation

##### Human dentate gyrus dissection, RNA extraction and cDNA synthesis

Human tissue was obtained from the Biobanc of the Hospital Clinic of Barcelona and their use approved by the Ethics Committee for Research with Drugs of Parc de Salut MAR (Comité de Ética de la Investigación con medicamentos del Parc de Salut MAR (CEIC-PSMAR); 2021/10159). Post-mortem hippocampi samples from Down syndrome patients and from age- and sex-matched unaffected controls (Supplementary Table 6) were embedded in Optimal cutting temperature compound (OCT) and cryosected. 30 μm sections were obtained and mounted on Superfrost charged slides and stored at - 80°C until being processed. To dissect the DG for RNA extraction, the slides were processed as explained in [105] for cresyl staining. The DG was identified histologically (Supplementary Fig. 5c) and dissected with the tip of a P1000 pipette. RNA concentrations ranged from 30 to 300 ng/μl. All RNA samples were subjected to DNase digestion with DNase I (Sigma #AMPD1– 1KT). Single-stranded cDNA was synthesized from 1000 ng of total RNA using SuperScript^™^ III Reverse Transcriptase and oligo(dT) primers (Thermo Scientific #18080093) following the manufacturer’s instructions.

##### Quantitative Real-time PCR in the human dentate gyrus

We validated the downregulation of Snhg11 by RT-qPCR. Briefly, RT-qPCR reactions were carried out using LightCycler^®^ 480 SYBR Green I Master Mix (Roche #04-887- 352-001) on a LightCycler^®^ Real-Time PCR System (Roche). The final volume for each reaction was 20 μl with 500 nM of corresponding gene specific primers (Snhg11 forward primer: tgggagttgtcatgttggga, Snhg11 reverse primer: actcgtcactcttggtctgt, Gapdh forward primer: attccacccatggcaaattc, Gapdh reverse primer: gggatttccattgatgacaagc), and 5 μl of total cDNA diluted 1:5. A negative water control and a no-RT negative control was included in each run. The thermal cycling was initiated at 95 °C for 10 minutes followed by 45 cycles of 10 s at 95 °C and 15 s at 55°C, the optimal annealing temperature for our target genes. Melting curve analyses were carried out at the end of each run of qPCR to assess the production of single, specific products.

##### Dp16 dentate gyrus RNA extraction and cDNA synthesis

Dp16 dissected dentate gyri were received from Marie-Claude Potier lab. For RNA extraction, frozen tissues were homogenized in 1mL of TRIzol. Afterwards, 200μl of chloroform was added and incubated for 5 min. Then, the samples were centrifuged at 12,000g for 15 min at 4 °C, and the upper aqueous phase was transferred to a new tube. RNA was precipitated with 2× volume of molecular-grade isopropanol. The samples were then incubated for 10 min at RT and centrifuged at 12,000g for 10 min at 4 °C. The pellet was then washed with 75% ethanol and resuspended with nuclease-free water after air drying for 5 min on the benchtop. RNA purity was assessed using a NanoDrop 2000 (Thermo Scientific #ND-2000). RNA concentrations ranged from 30 to 200 ng/μl. All RNA samples were subjected to DNase digestion with DNase I (Sigma #AMPD1– 1KT). Single-stranded cDNA was synthesized from 1000 ng of total RNA using SuperScript^™^ III Reverse Transcriptase and oligo(dT) primers (Thermo Scientific #18080093) following the manufacturer’s instructions.

##### Quantitative Real-time PCR in the Dp16 dentate gyrus

cDNA synthesis and quantitative Real-time PCR was performed as described above. In this case, the 500 nM of corresponding gene specific primers (Snhg11 forward primer: ctgtccagctaggaagcagc, Snhg11 reverse primer: tcgctccacactgatgttgg, Ntng1 forward primer: aggggcaagagaccaagg, Ntng1 reverse primer: agggatggtgtctatcgtcct, Gapdh forward primer: ggagattgttgccatcaacga, Gapdh reverse primer: tgaagacaccagtagactccacgac) were used.

#### ASO-mediated knockdown of Snhg11

##### Antisense oligonucleotides

ASOs (IDT) were uniformly modified with 2′-O-(2-methoxy) ethyl sugars (2′MOE), phosphorothioate backbone, and 5′-methyl cytosine. ASOs were dissolved in 0.9% saline to a concentration of 75 μg/μL. ASO sequences are provided in Supplementary Table 4. Both ASO sequences were subjected to a BLAST search. The Snhg11-ASO had only positive matches for the target sequences and no other mouse coding sequence. The control ASO (Control-ASO), which included the same nucleotides in a scrambled order, did not generate any full match to identified gene sequences in the database.

#### In vitro knockdown of Snhg11

##### Cell culture and ASO transfection

Mouse N2a neuroblastoma cells were maintained in high glucose Dulbecco’s modified Eagle’s medium (Thermo Fisher #11965084) supplemented with 10% FBS, 100 U/mL penicillin and 100ug/mL streptomycin in a humid atmosphere containing 95% air and 5% CO_2_ at 37°C. Snhg11-ASO and Control-ASO were transfected into N2a cells using Lipofectamine 3000 (Thermo Fisher #L3000001) in a 96-well format. For each well, 1.5 μL of the Snhg11-ASO or Control-ASO were diluted in 8.5uL OptiMEM medium (Thermo Fisher #31985062), 0.3 μL P3000 and 0.25 μL Lipofectamine and incubated at room temperature for 15 minutes. Meanwhile, N2A cells were trypsinized, counted and diluted to 22000 cells/140 μL with antibiotic-free DMEM supplemented with 10% FBS. After incubation, 10 μL of the ASO-lipid complex was added to each well in triplicate followed by 140μL of the diluted cells for a final volume of 150 μL.

##### RNA extraction from N2a and Quantitative Real-time PCR

To ensure *Snhg11* knockdown, 72 hours after being transfected, N2a cells were washed with pre-warmed PBS and trypsinized. Cells were then pelleted at 300g for 7 minutes and resuspended in 200μL of Homogenization Buffer from the Maxwell RSC simplyRNA Tissue Kit (Promega #AS1340). Cells were then lysed by adding 200 μL of lysis buffer and transferred into the Maxwell RSC Cartridge. Cartridges were then loaded into the Maxprep Liquid Handler and RNA was extracted in 30 μL of water following the simplyRNA Cells protocol provided by the manufacturer. cDNA synthesis and quantitative Real-time PCR was performed as described above.

##### CCK8 proliferation assay

Snhg11-ASO and Control-ASO transfected N2a cells were seeded into 96-well plates overnight. At 24, 48, or 72 h post-transfection, 10 μl Cell Counting Kit-8 solution (MedChem Express HY-K0301) was added into each well of 96-well plates, followed by further incubation of 3 h at 37 °C. Then, the absorbance was determined at a wavelength of 450 nm to assess cell proliferation.

##### Colony Formation Assay

*Snhg11*-ASO and Control-ASO transfected cells were plated in 6-well plates at 500 cells/well in triplicate. The cells were maintained in cell culture with media change every 3 days. After 21 days, they were washed 3 times with PBS, fixed with 4% PFA for 10 minutes, stained with a 0.06% crystal violet solution for 1 hour at 37°C and colonies counted under an inverted microscope.

#### In vivo knockdown of Snhg11

##### Stereotactic injection

For *in vivo* injection of ASOs, the same wild type mice littermates of Ts65Dn were used. Briefly 4 month old male and female mice (n =16 per experimental condition) were anesthetized by intraperitoneal injection of ketamine (75 mg/kg) and medetomidine (10 mg/kg) and placed on a stereotaxic frame. For each injection, a small incision was made, the skull was exposed, and a small burr hole was drilled at the proper coordinates (−2.2 mm anteroposterior, +/−1.3 mm mediolateral and −2 mm dorsoventral, relative to the bregma). Three minutes after the needle (Hamilton #65458–01) was placed into the proper coordinates, a total of 660nL of saline-diluted Snhg11-ASO or Control-ASO at a concentration 75 μg/μl was delivered into each hemisphere of the DG at an infusion rate of 50nL/minute using an injection pump. After the infusion was completed, the needle was kept in the same position to ensure no contamination of other brain areas with residual volume. The incision site was sutured using surgical glue (Cemave #1050052) and the mouse was allowed to recover in a temperature-controlled environment. Following surgery, buprenorphine was administered at 0.1 mg/kg/day for 3 days. During this time, weight, grooming activity and home cage activity were controlled. In the first round of experiments, the site of injection in the dorsal DG was confirmed by the injection of 1 μL of trypan blue in anesthetized mice.

##### Hippocampi isolation, RNA extraction, cDNA synthesis and quantitative Real-time PCR

To ensure knockdown of *Snhg11*, three days after the stereotactic infusion, the hippocampus of injected mice (N=3 per group) was dissected, separated into two equal halves corresponding to the dorsal and ventral halves, with the help of an scapel [106], snap-frozen in liquid nitrogen and kept at −80°C until processed. For RNA extraction, frozen tissues were homogenized in 1mL of TRIzol. Afterwards, 200μl of chloroform was added and incubated for 5 min. Then, the samples were centrifuged at 12,000*g* for 15 min at 4 °C, and the upper aqueous phase was transferred to a new tube. RNA was precipitated with 2× volume of molecular-grade isopropanol. The samples were then incubated for 10 min at RT and centrifuged at 12,000*g* for 10 min at 4 °C. The pellet was then washed with 75% ethanol and resuspended with nuclease-free water after air drying for 5 min on the benchtop. RNA purity was assessed using a NanoDrop 2000 (Thermo Scientific #ND-2000). RNA concentrations ranged from 30 to 200 ng/μl. All RNA samples were subjected to DNase digestion with DNase I (Sigma #AMPD1–1KT). Single-stranded cDNA was synthesized from 1000 ng of total RNA using SuperScript^™^ III Reverse Transcriptase and oligo(dT) primers (Thermo Scientific #18080093) following the manufacturer’s instructions. Quantitative Real-time PCR was performed as described above.

##### RNA-sequencing

The hippocampal RNA was also used for RNA-seq. Library preparation for mRNA sequencing was performed according to Illumina standard protocols (TruSeq, Illumina) from ribo-depleted total RNA. Libraries were quality-controlled and quantified using Nanodrop 2000 (Thermo Scientific), Agilent 2100 Bioanalyzer (Agilent Technologies) and Qubit (Life Technologies). Samples were sequenced (paired end, 50 base pairs in length) using an Illumina HiSeq2500 apparatus with a depth of at least 50 million reads.

Reads were then aligned using STAR (v.2.5.2a) [107] to reference genome (mm10) and quantified using Rsubreads (v.1.26) [108]. Gene expression data was visualized by PCA plot and one Snhg11-ASO sample was identified as an outlier and removed for downstream analyses. Differential expression analysis was performed using DESeq2 (v.1.10.0) [109]. Genes with FDR values of <0.05 were considered significantly changed.

##### Gene set enrichment

The significantly downregulated genes in Snhg11-ASO injected animals were tested for enriched Gene Ontology processes, using a hypergeometric test (shinyGO [102]), and corrected for multiple hypotheses by FDR. Processes with p adjusted value < 0.05 were reported as significantly enriched. The complete list of genes present in the dataset was used as the universe for the hypergeometric test.

#### Direct RNA nanopore sequencing

##### DRS library preparation

400 ng of DNase-treated total RNA from 2 biological replicates of Control-ASO and Snhg11-ASO hippocampi were polyadenylated with E. coli PolyA Polymerase (NEB #M0276L) for 15 min at 37°C. Samples were then cleaned with RNAClean XP beads (Beckman Coulter, #A63987). Then, 50 ng from each sample were ligated to 4 pre-annealed RT adaptors for multiplexing (sequence and protocol from Smith et al. 2020), using concentrated T4 DNA Ligase (NEB, #M0202T). Samples were then prepared for sequencing using the ONT Direct RNA Sequencing protocol version DRS_9080_v2_revI_14Aug2019 and using theSQK-RNA002 kit, except for the reverse transcription step, in which SuperScript IV RT (Thermo Fisher Scientific, 18090010) at 50°C for 15 min was used, followed by heat inactivation at 70°C for 5 min. The reverse-transcribed RNA was purified with 1.8X RNAClean XP beads and washed with 70% freshly prepared ethanol. After cleanup, 50 ng of reverse transcribed RNA from each sample were pooled to reach 200 ng for ligation with the RMX adapter. The mix was purified using 1X RNAClean XP beads according to SQK-RNA002 protocol. The sample was then eluted in Elution Buffer (EB) and mixed with RNA Running Buffer (RRB), and loaded onto a primed R9.4.1 flowcell. The sequencing was run on a MinION sequencer with MinKNOW acquisition software version 22.03.5.

##### DRS data pre-processing

Raw fast5 files were processed with the Master of Pores [110] (version 2 of the pipeline, which is publicly available in GitHub (https://github.com/biocorecrg/MOP2). The mop_preprocess module was used to process the samples, using DeePlexiCon [111] for demultiplexing of the raw fast5 files, Guppy basecaller 3.4.5. (https://nanoporetech.com) for base calling, and graphmap version 0.3.0 [112] for mapping with default parameters. Reads were mapped to a custom mouse rRNA FASTA reference obtained from NCBI nucleotide database (entries: 5S rRNA - NC_000074.6; 5.8S rRNA - NR_003280.2, 18S rRNA - NR_003278.3, 28S rRNA - NR_003279.1).

##### Analysis of RNA modifications in direct RNA sequencing data

Base quality, mismatch frequency, insertion frequency and deletion frequency for each position of each transcript were computed with Epinano [113] version 1.2 (https://github.com/novoalab/EpiNano) from the bam files. The difference in mismatch frequency was calculated for comparison between Control-ASO and Snhg11-ASO samples in a pairwise manner.

#### Memory tests

We performed a novel object recognition test (NOR), and an Object Pattern Separation (OPS) test for testing hippocampal function and Delay fear-conditioning (DFC) for non-hippocampal memory. For the NOR and OPS tests, four groups of animals were used: WT, WT injected with Snhg11-ASO, WT injected with Control-ASO and TS. 16 animals per group (8 males and 8 females) were used. For DFC, only WT injected with Snhg11-ASO (N=6 males and 5 females) and WT injected with Control-ASO (N=7 males and 5 females) were used. In the case of injected animals, they were let to recover for 5–7 days before being subjected to any task. Three days before testing mice were handled by the experimenter. All the behavioral experiments were conducted in 4 month old male and female mice during the light cycle (7:30 am to 13:00 pm). All mice were individually handled and habituated to the investigator for five minutes, in three separate days. Handling took place in a different room where the behavioral apparatus was located. Before each handling session, mice were transported to the handling room by a wheeled trolley to habituate them to the journey.

##### Novel Object Recognition

The novel object recognition test (NOR), is a relatively fast and efficient means for testing different phases of learning and memory in mice. The test was conducted as explained in previously [114]. Briefly, the NOR protocol consists of four phases, namely habituation, familiarization, short-term test and long-term sessions. During the habituation session, mice were allowed to freely explore the empty open field for 10 minutes. 24 hours later, each mouse was returned to the arena containing two identical objects placed at symmetrical positions 5 cm from the arena wall and allowed to explore them freely for 15 minutes. During the familiarization session most mice reached a minimum exploration for each object of 30 seconds. Mice not reaching this threshold, were excluded from the analysis. After a retention interval of 1 hour, the mouse was returned to the arena in which one of the objects was replaced by a novel object and let to explore both the familiar and the novel object for 5 minutes. Similarly, 24 hours later the animal was returned to the arena in which the novel object of the short-term session was replaced by a third object validated for achieving similar levels of exploration. The animal was then let to explore both objects for 5 minutes. All sessions were video-taped.

##### Object Pattern Separation (OPS)

To test pattern separation memory, the protocol described by van Hagen et al. (2015) [56][44], was followed with few modifications. The same square chamber used for NOR was used for OPS. Two days after completing the NOR, mice were habituated again to the open field without any objects for 10 minutes. 24 hours later, mice were presented with two identical objects placed in two symmetrical spots 5 cm from the arena wall. The objects were made with building blocks stuck to the arena floor so that mice were not able to move them. Animals were let 6 minutes to explore. The test session was performed 1 hour later, where one of the objects was placed in a novel location inside the arena, 8 cm away from the initial location. Mice were let 4 minutes to explore. All sessions were video-taped.

##### NOR and OPS behavioural experimental variables

Locomotor activity was quantified as the total distance traveled in the apparatus during the experimental sessions. Thigmotaxis refers to the disposition to remain close to the walls of the apparatus. It is measured as distance traveled or percentage of time spent in the periphery of the apparatus. It decreases gradually during the first minutes of exploration, and can be used as an index of anxiety [115]. Exploration time is defined as the action of pointing the nose toward an object, at a maximum distance of 2 cm or touching it. Going around the objects or sitting on the object is not evaluated as exploration time. As such, the exploration time is only computed when the snout of the animal is directed toward the object, sniffing or touching it. Discrimination index (DI), is the most relevant parameter in the NOR and OPS, which is a measure of the recognition memory of the animal. It is calculated as the difference in exploration time for the familiar versus the novel object in the case of NOR, or the familiar position versus the novel position in the OPS, divided by the total amount of exploration.

##### *Delay fear-conditioning* (DFC)

Snhg11-ASO or Control-ASO injected mice were used. Two different contexts were employed in two different cages. Context A consisted in a chamber (29 X 25 X 22 cm) with Perspex floors and transparent circular ceilings and Context B chamber (30 X 25 X 33cm) had grid floors, and opaque square ceilings. Context A was scented with ethanol 70% while context B was scented with 1% acetic acid. Mice were trained in Context B in a session that lasted 180s. Mice explored the context for 120s (pre-tone). Then a tone (85 dB, 2800 Hz) was delivered for 30s (tone). Immediately after the tone, a single shock was delivered for 2s (0.6 mA) and mice remained in the cage for 30s (post tone). After training, mice were placed back in their home cages and transported to the holding room. Testing was performed in Context A. This time, no shocks were delivered. During 120s (pre-tone) mice explored the context. Then the same tone as in the training (85dB, 2800 Hz) was applied for 60s (tone). After the tone, mice remained in the cage for 30s. It is important to note that mice generally showed little freezing when first placed in Context A (before the tone was presented) and that there was no difference between groups in these pre-tone (or baseline) levels of freezing. Freezing behavior (>800ms immobility) was automatically detected by Packwin 2.0 software (Panlab, Harvard Apparatus). Cages were calibrated according to manufacturer instructions each day of experiment.

##### BrdU assay

Adult neurogenesis was measured by the incorporation of the thymidine analogue BrdU, which is incorporated into the DNA of dividing cells in detectable quantities during the S phase of cell division. 4 month old wild type male mice were stereotaxically infused as previously described with Control-ASO (N = 3) or Snhg11-ASO (N = 3). After one week, each animal received four intraperitoneal injections of BrdU (Merck #B5002) at 50mg/kg every 2 hours on a single day [116, 117]. Animals were sacrificed by suffocation with carbon dioxide (CO2) 24 hours after the last injection and transcardially perfused with 50mL of chilled PBS followed by fixation with 50–100mL of 4% (PFA). After perfusion, brains were removed and post-fixed overnight in 4% paraformaldehyde at 4°C. Sections 40 μm thick were then prepared in the coronal plane using a vibratome. From each animal, every sixth section (240 μm apart) of the dorsal hippocampus (−1.3 mm posterior to bregma through 2.2 mm posterior to bregma)was used for BrdU Immunofluorescence. Sections were rinsed three times with PBS and incubated in 2M HCl for 15 minutes at 37°C to denature DNA and allow the access for the anti-BrdU antibody. The acid was then neutralized by rinsing the sections with PBS. Sections were incubated with blocking solution (10% Donkey serum in TBS-Tween 0.5%) for 1h at room temperature and with the BrdU primary antibody (1:300; MBL, MI-11–3) diluted in blocking solution for 24 hours at 4°C on a shaker. Sections were rinsed again with PBS and incubated with the secondary antibody (1:500; Donkey anti-mouse 488, Jackson #715- 545-150) diluted in an incubation buffer (TBS-Tween 0.5% with 5% Donkey serum) for 3 hours at room temperature on a shaker. Rinsed sections were then mounted on uncoated Superfrost slides and covered with antifade and coverslip. For visualization and photography, specimens were observed under a confocal SP5 microscope. For neurogenesis quantification, 488+ cells located in the subgranular zone were counted and the whole granule cell layer area measured to determine density.

#### In vitro electrophysiology

##### Slice preparation

4 month old wild type mice were stereotaxically infused as previously described with Control-ASO (N = 4) or Snhg11-ASO (N = 5). After 5–7 days mice were decapitated and brains were extracted. Sagittal brain slices (400 μm) were prepared using a vibratome (VT1200S, Leica Microsystems, Nussloch, Deutschland) and incubated for one hour at room temperature in artificial cerebrospinal fluid (aCSF) that contained 124 mM NaCl, 2,5 mM KCl, 1,25 mM KH2PO4, 1 mM MgSO4, 26 mM NaHCO3, 2mM CaCl2 and 10 mM glucose, and gassed with a 95% O2/ 5% CO2 mixture at pH 7,3–7,4. Slices were transferred individually to an immersion chamber and perfused with oxygenated aCSF containing picrotoxin (50 μM, Sigma-Aldrich) at 2ml/min warmed at near physiological temperature (31 ± 2°C).

##### Recording of evoked field postsynaptic potentials

Field postsynaptic potentials (fEPSPs) were recorded using glass capillary electrodes (4 – 5 MΩ) filled with ACSF positioned in the medial molecular layer of the upper blade of the dentate gyrus. Evoked fEPSPs were elicited by stimulation of the perforant pathway with an extracellular bipolar tungsten electrode via DS3 Isolated Current Stimulator (Digitimer), that was set to deliver monophasic of 50 μs duration.

##### Long-term potentiation

At the beginning of each experiment input/output (I/O) relationships were obtained increasing progressively the intensity of stimuli, reaching a maximal fEPSP response. In order to perform LTP experiments, the stimulus intensity was adjusted to elicit 50–60% of the maximum response signal and kept constant throughout the experiment. Data were stored through an acquisition system Multiclamp 700B (Axon Instruments) and were digitized at 16 bits (Axon 1550B Digidata, Molecular Devices) using pClamp10 (Molecular Devices). After recording stable baseline responses for 10 min, LTP was induced with four trains of theta burst stimulation (TBS; 10 bursts of 5 pulses at 100 Hz, with an interval of 200 ms between bursts) with 30 seconds between trains. After induction, the responses were recorded every 15 seconds during 1 hour. Changes in the fEPSP slope were calculated in relation to the baseline fEPSP responses during the last 10 minutes before TBS and the time course of LTP values was then normalized to this baseline. Experiments where the recording during the basal conditions was unstable during 30 min were discarded and experiments where the response after the tetanization felt more than the 80% of the basal response were also discarded. The statistical significance of LTP induction within each group was determined using an unpaired Student’s t test between mean fEPSPs slopes recorded 50 minutes after LTP induction during 10 minutes. We limited the number of slices per animal, performing the experiment in a maximum of three slices per animal in order to avoid over-representation of an animal Data were analyzed using Clampfit 9. Signals were filtered using a lowpass filter (Bessel 8-pole) with a −3dB cutoff of 500 Hz and fEPSP responses were promediated to obtain a response every 30 seconds in order to reduce noise.

### Statistical analyses of behavioral, histological and qPCR experiments

In the cases where more than two groups were compared, either a one or two-way ANOVA was conducted depending on the number of factors (Group and Sex) to investigate statistical differences in experimental variables. For pairwise comparisons, the Saphiro-Wilk and Bartletttests were used to assess normality of values and homogeneity of variances between groups, respectively. When the two conditions were met, pairwise differences were tested by Tukey’s *post hoc* test.

When only two conditions were compared, statistical differences were tested by Student’s t-test after confirming normality and homogeneity of variances between groups by Shapiro-Wilk test and Fisher’s F test, respectively.

In the cases where distribution of the data could not be estimated due to the small sample size, statistical differences were assessed by a permutation test.

All statistical analyses were two-tailed. P-values were considered to be significant when α < 0.05.

## Data Availability

The snRNA-seq and bulk RNA-seq data that support the findings of this study have been deposited in the Gene Expression Omnibus repository, with the series record GSE212351 and GSE212258. Basecalled FAST5 of nanopore direct RNA sequencing runs have been deposited to ENA, under accession code PRJEB58921. All the raw data and supplementary materials are available upon request.
